# Risk-adjusted trifecta outcomes in ultrasound-guided RFA of T1a renal masses: experience from a large tertiary cancer center

**DOI:** 10.1590/S1677-5538.IBJU.2025.0034

**Published:** 2025-04-15

**Authors:** Derun Li, Jiyu Yang, Xiang Wang, Yi Liu, Gangzhi Shan, Zibo Zhang, Xu Han, Zhihua Li, Xuesong Li

**Affiliations:** 1 Peking University Institute of Urology Department of Urology, Peking University First Hospital Beijing China Department of Urology, Peking University First Hospital, Institute of Urology, Peking University, National Urological Cancer Centre, Beijing, China; 2 Peking University Peking University First Hospital Department of Nursing Beijing China Department of Nursing, Peking University First Hospital, Peking University, Beijing, China

**Keywords:** Kidney Neoplasms, Radiofrequency Ablation, Recurrence

## Abstract

**Purpose:**

To evaluate the trifecta outcomes of ultrasound-guided radiofrequency ablation (RFA) of T1a renal masses and to identify factors influencing trifecta outcomes.

**Materials and Methods:**

We retrospectively reviewed data from patients who underwent ultrasound-guided RFA at Peking University First Hospital between March 2017 and May 2024. Baseline demographics, perioperative outcomes and follow-up results were collected. The trifecta outcomes were defined as the absence of severe complications, incomplete ablation and tumour recurrence. Multivariate logistic regression analysis was performed to identify risk factors for trifecta failure.

**Results:**

Among 270 patients (140 left-sided and 130 right-sided), the median tumour size was 1.97 (range 0.80-3.86) cm, and 32 (11.9%) patients had a history of ipsilateral partial nephrectomy. During the median follow-up of 35.6 (range 6.2-91.4) months, the rates of severe complications, tumour recurrence, and incomplete ablation were 1.1%, 7.4%, and 7.4%, respectively. The trifecta outcome was achieved in 227 (84.1%) patients. Multivariate analysis revealed that tumour size [odds ratio (OR): 2.144, p = 0.007] and history of ipsilateral partial nephrectomy (OR: 3.894, p = 0.002) independently predicted trifecta failure.

**Conclusion:**

Ultrasound-guided RFA is a safe and effective treatment for T1a renal masses. Tumour size and a history of ipsilateral partial nephrectomy were significantly associated with trifecta failure.

## INTRODUCTION

Renal masses are among the most common tumours of the urinary system, with the majority being malignant. Renal cell carcinoma (RCC), the most prevalent malignant kidney tumour, accounted for an estimated 434,840 cases globally in 2022 (1). RCC predominantly affects individuals aged 60-70 years, with a male-to-female ratio of 3:2 (2). In the United States, RCC ranks as the sixth most common cancer in males and the ninth most common cancer in females (1). Early diagnosis and surgical intervention are critical for improving patient prognosis.

The World Health Organization (WHO) has classified more than 20 subtypes of RCC (3), with clear cell carcinoma (75-80%), papillary carcinoma (10-15%), and chromophobe carcinoma (5%) being the most prevalent pathological types (4). Treatment strategies for renal masses are tailored according to disease stage, with options ranging from radical nephrectomy, partial nephrectomy to minimally invasive techniques.

Currently, radiofrequency ablation (RFA) and microwave ablation have emerged as the primary minimally invasive modalities for small renal masses (5). RFA, a heat-mediated tissue destruction technique, was initially employed for treating primary and metastatic liver tumours (6). This approach is distinguished by its precise action and ability to create larger ablation zones within shorter treatment durations (7). With technological advancements, RFA has been extended to the management of various tumour types, including stage T1a renal masses, where it has demonstrated potential for achieving complete tumour ablation. Although the safety and efficacy of RFA have been confirmed by numerous studies (8, 9), further research is still needed to optimize strategies for reducing complications and improving patient outcomes. Additionally, the identification of factors associated with complications and long-term prognosis remains an understudied area. In this context, a trifecta of outcomes, a composite endpoint encompassing the absence of severe complications, tumour recurrence, and incomplete ablation, has emerged as a valuable tool for assessing the prognosis of surgical patients (10–13). Based on extensive clinical experience in managing T1a renal masses with ultrasound-guided RFA, we observed that trifecta outcomes may be influenced by specific clinical factors. Notably, tumour size and a history of ipsilateral partial nephrectomy emerged as potential predictors of trifecta failure. The aim of this study was to evaluate the trifecta outcome for patients with T1a renal masses who underwent ultrasound-guided RFA and to identify risk factors influencing these outcomes.

## MATERIALS AND METHODS

### Study population

**Figure 1:**
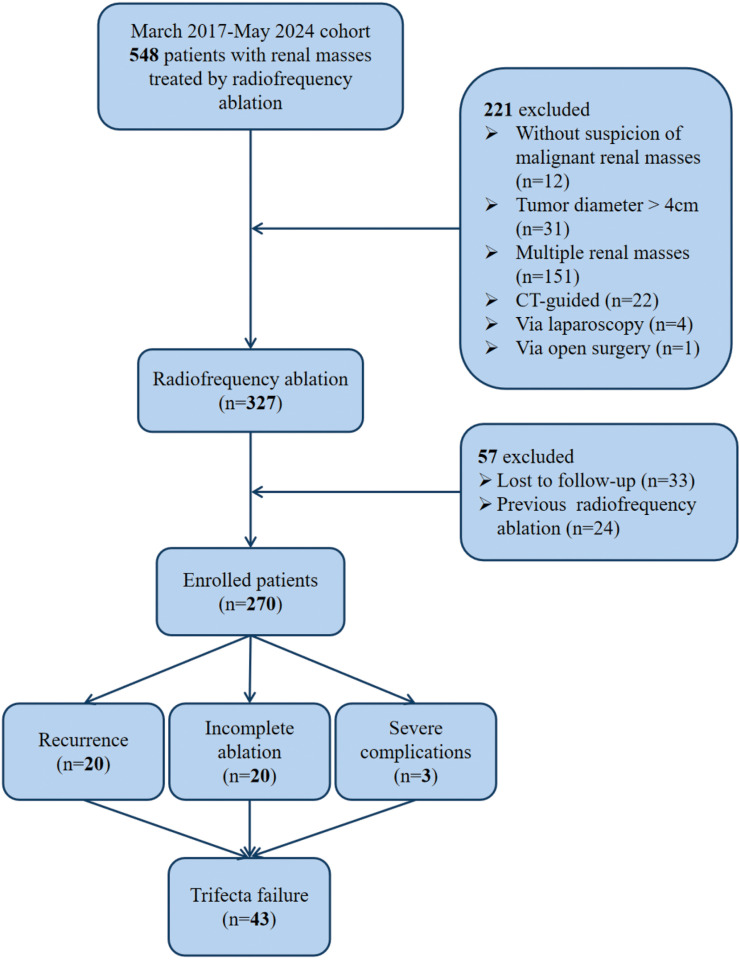
STROBE Diagram of Study Enrollment and Final Analysis Cohort.

We retrospectively collected data from patients who underwent ultrasound-guided RFA for T1a renal masses at Peking University First Hospital between March 2017 and May 2024. This study followed the Strengthening the Reporting of Observational Studies in Epidemiology (STROBE) reporting guidelines. Informed consent was obtained from all subjects who met the inclusion criteria and agreed to participate in the study, which was carried out in accordance with the Principles of the Declaration of Helsinki. Finally, a total of 270 patients were included according to the inclusion and exclusion criteria (Figure-1).

### Inclusion Criteria:

1. Patients with suspicion of malignant renal masses based on preoperative imaging examinations as evaluated by multiple radiologists;
2. Clinical stage of T1a;
3. Unilateral tumour masses;
4. Underwent ultrasound-guided RFA;

### Exclusion Criteria:

1. Patients scheduled to undergo cytoreductive surgery;
2. Patients with a clinical stage of T1b or higher;
3. Patients with multiple or bilateral tumour masses;
4. Patients who underwent RFA via other approaches (open/laparoscopic/robotic surgery);
5. Patients who underwent RFA guided by CT or MRI;
6. Patients lost to follow-up;
7. Patients with a history of prior RFA.

### Data Collection

We collected data on baseline characteristics, including sex, age, body mass index (BMI), history of ipsilateral partial nephrectomy, serum creatinine (Scr), and estimated glomerular filtration rate (eGFR). The eGFR was calculated via the chronic kidney disease epidemiology collaboration (CKD-EPI) equation. The clinical characteristics included tumour size and laterality. The procedure details included the type of anaesthesia, procedure time, power and cumulative energy. All samples were classified by histopathological examination and graded according to the 2016 WHO guidelines (14). Postoperative complications were graded on the basis of the Clavien-Dindo (CD) system, with grades III and IV considered major (15).

### Biopsy and Radiofrequency Ablation Procedure

Biopsies and RFA were performed by experienced operators under ultrasound guidance. The type of anaesthesia, including general (0.7%, n = 2), local (94.4%, n = 255), strengthening local (4.4%, n = 12) and monitored anaesthesia care (0.4%, n = 1), was selected based on the preoperative evaluation. Biopsy procedures involved obtaining multiple tissue core samples to increase diagnostic accuracy, with real-time confirmation from pathologists. Patient positioning was determined by tumour location, and needle selection (T20, T30, or combined) was based on tumour size. Ablation parameters, including power, duration, and energy, were adjusted according to intraoperative ultrasound findings, tissue impedance, and tumour dimensions.

**Figure 2:**
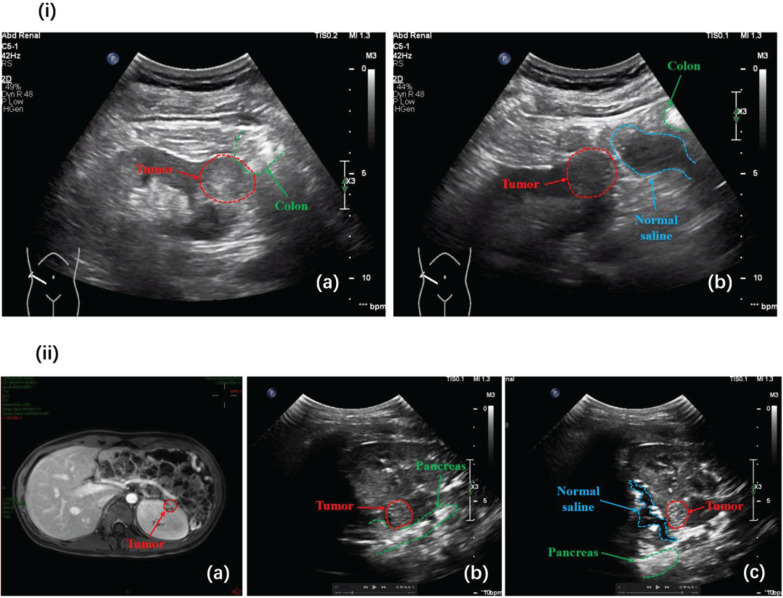
Schematic Illustration of Hydro-dissection for Protecting Adjacent Tissues and Organs during Radiofrequency Ablation.

During our surgical procedure, several critical steps were meticulously executed. First, our current surgical protocol involves performing RFA immediately after biopsy without awaiting pathological results. This approach was adopted based on the rationale that the ablation needle can be inserted directly along the biopsy tract during the ablation process, thereby minimizing additional surgical trauma and reducing associated costs. Furthermore, simultaneous ablation of the needle tract upon withdrawal helped mitigate the potential risks of needle tract implantation and metastasis. Second, for ventral or lower pole tumours, hydro-dissection was employed, involving the injection of normal saline into the renal fascia to protect adjacent tissues and organs, as illustrated in Figure-2. Finally, routine ultrasound was performed immediately after ablation to evaluate the completeness of the ablation area.

(i) a. Ultrasound imaging demonstrated the tumor adjacent to the colon; b. Saline injection created a protective barrier, achieving safe separation between the tumor and colon.

(ii) a. MRI confirmed the anatomical location of the tumor; b. Ultrasound imaging showed the tumor in close proximity to the pancreas; c. Saline injection isolated the tumor from the pancreas, preventing thermal injury.

### Follow-up

Follow-up assessments were scheduled within 3 months post-surgery, with different imaging methods (enhanced CT, enhanced MRI, or contrast-enhanced ultrasound). For patients with renal insufficiency, ultrasound contrast was used for imaging. Complete tumour ablation was defined as the absence of residual tumour within the ablation zone. If complete tumour ablation was achieved, follow-up evaluations were routinely conducted at 6 months and 1 year postoperatively, followed by annual assessments. Incomplete ablation was defined as persistent contrast enhancement at the original tumour site on follow-up imaging (CT, MRI, or contrast-enhanced ultrasound). Tumour recurrence was diagnosed based on new enhancing lesions detected outside the ablation zone. Trifecta outcomes were defined as the absence of severe complications, tumour recurrence, and incomplete ablation.

## Statistical Methods

Continuous variables are presented as the means ± standard deviations for normally distributed data and medians (interquartile ranges) for non-normally distributed data. Categorical variables are presented as frequencies and percentages and were compared via chi-square tests. Paired Wilcoxon rank tests were used to assess changes in the Scr level and eGFR from the pre- to postoperative periods. Univariate and multivariate logistic regression were performed to identify prognostic factors associated with trifecta outcomes. Variables with theoretical clinical importance and those achieving p < 0.10 in the univariate analysis were included in the multivariate model. Statistical analyses were performed via SPSS (version 27.0; IBM, Armonk, New York), with significance set at p < 0.05.

## RESULTS

### Baseline Characteristics

A total of 270 patients were included in this study. The demographic and clinical characteristics are summarized in Table-1. The median age was 63 years (IQR: 54-72) and most subjects were male, with 182 male patients (67.4%). The mean BMI was 25.32 kg/m2 (SD 3.86). The median baseline Scr was 85.05 μmol/L (IQR: 72.68-103.70), and the eGFR was 83.44 mL/(min*1.73 m2) (IQR: 65.10-98.46). All patients presented with unilateral lesions, including 140 right-sided and 130 left-sided tumours. The median tumour size was 1.97 cm (IQR: 1.60-2.41). Thirty-two (11.9%) patients had a history of prior ipsilateral partial nephrectomy. Additionally, 14 patients (5.2%) had a solitary kidney. With respect to tumour location, 189 cases (70.0%) were exophytic, and 81 cases (30.0%) were endophytic.

**Table 1:** Baseline Characteristics and Perioperative Outcomes.

Variables			Value
**Sex, n (%)**			
		Male	182 (67.4)
		Female	88 (32.6)
Age, years, median (IQR)			63 (54-72)
BMI, kg/m^2^, mean (SD)			25.32 (3.86)
Scr, μmol/L, median (IQR)			85.05 (72.68-103.70)
eGFR, mL/min/1.73m2, median (IQR)			83.44 (65.10-98.46)
**Laterality, n (%)**			
		Left	140 (51.9)
		Right	130 (48.1)
Prior Ipsilateral Partial Nephrectomy, n (%)			51 (18.9)
Solitary Kidney, n (%)			14 (5.2)
**Exophytic Appearance, n (%)**			
		Yes (Exophytic)	189 (70.0)
		No (Endophytic)	81 (30.0)
Tumor Size, cm, median (IQR)			1.97 (1.60-2.41)
**Anesthesia Methods, n (%)**			
		General	2 (0.7)
		Local	255 (94.4)
		Strengthening Local	12 (4.4)
		MAC	1 (0.4)
Ablation Power, watts, median (IQR)			40 (20-40)
Ablation Time, minutes, median (IQR)			20.5 (18.0-24.5)
Cumulative Energy, KJ, median (IQR)			22.50 (17.80-30.40)
**Biopsy Histology**			
	**Malignant, n (%)**		
		Clear Cell RCC	174 (64.4)
		Papillary RCC	11 (4.1)
		Chromophobe RCC	6 (2.2)
	**Benign, n (%)**		
		Oncocytoma	10 (3.7)
		Angioleiomyolipoma	16 (5.9)
	* **Other, n (%)**		53 (19.6)
**Complications, n (%)**			
		CD Grade I	9 (3.3)
		CD Grade II	4 (1.5)
		CD Grade IIIa	1 (0.4)
		CD Grade IVa	1 (0.4)
		CD Grade V	1 (0.4)

* Indeterminate renal cell carcinoma, non-neoplastic benign tissue, or no biopsy performed.

IQR = Interquartile Range; BMI = Body Mass Index; SD = Standard Deviation; Scr = Serum Creatinine; eGFR = Estimated Glomerular Filtration Rate; MAC = Monitored Anesthesia Care; RCC = Renal Cell Carcinoma; CD = Clavien-Dindo.

### Perioperative outcomes

The perioperative outcomes and postoperative complications are detailed in Table-1. The median RFA power was 40 watts (IQR: 20-40), with a median procedure time of 20.5 minutes (IQR: 18.0-24.5; range 8.0-47.5). The median cumulative energy delivered was 22.50 KJ (IQR: 17.80-30.40). The median hospitalization duration was 2 days (IQR: 2-4).

Postoperative pathological results revealed that 26 cases were benign (9.6%), with renal angiomyolipoma being the most common (5.9%, n = 16). Malignant cases accounted for 191 cases (70.7%), predominantly clear cell carcinoma (64.4%, n = 174). The remaining 53 cases (19.6%) included renal cell carcinoma with uncertain pathology (n = 12), non-neoplastic benign tissue (n = 21), or cases where biopsy was not performed (n = 20). Our data indicated that patients experienced a mild decline in renal function following radiofrequency ablation. For the 256 patients whose data were available, the Scr increased from a preoperative median of 85.34 μmol/L (IQR: 73.19-105.35) to a postoperative median of 88.28 μmol/L (IQR: 73.85-105.75), p = 0.007, and the eGFR decreased from a preoperative median of 83.44 mL/(min*1.73 m2) (IQR: 63.29-97.28) to a postoperative median of 79.86 mL/(min*1.73 m2) (IQR: 63.33-95.03), p = 0.004.

Complications were recorded in 16 patients (5.9%), with 3 patients (1.1%) experiencing severe complications. Among those three patients, the first patient (grade IIIa) developed gross haematuria secondary to arteriovenous fistula formation, which was successfully managed with interventional embolization. The second patient (grade IVa) presented with a perirenal hematoma complicated by acute renal insufficiency, necessitating urgent haemodialysis, followed by gradual recovery of renal function. The third patient (grade V) experienced intraoperative heart failure (New York Heart Association class IV) accompanied by septic shock and haemorrhagic complications. Despite aggressive supportive measures, including vasopressor administration and broad-spectrum antibiotics, the patient ultimately succumbed to multiorgan failure.

### Follow-up outcomes

During the median follow-up of 35.6 months (IQR: 17.4-61.4), 19 deaths occurred, including 6 attributed to RCC. Tumour recurrence and incomplete ablation were observed in 20 patients (7.4%), as detailed in Figure-1. Further treatment, including a second RFA (n = 8), radical nephrectomy (n = 5), partial nephrectomy (n = 1), targeted/immunotherapy (n = 1), and renal transplantation or dialysis (n = 2), was administered to 17 patients with recurrent tumours. The remaining 3 patients with tumour recurrence did not receive additional therapy due to advanced age (≥ 80 years) or the detection of widespread metastatic disease, for which palliative care was deemed more appropriate. Among the 20 incomplete ablation patients, all patients underwent further treatment, including a second RFA (n = 19) and radical nephrectomy (n = 1). All 40 patients with tumour recurrence and incomplete ablation had malignant pathology, predominantly clear cell carcinoma (12.6%, n = 34), followed by papillary carcinoma (0.4%, n = 1). The remaining 3 patients (1.1%) had indeterminate renal cell carcinoma, non-neoplastic benign tissue, or no biopsy performed.

### Trifecta Outcomes

The trifecta outcomes were achieved in 84.1% of patients. Multivariate logistic regression analysis (Table-2) revealed that tumour size [odds ratio (OR): 2.144, p = 0.007] and a history of ipsilateral partial nephrectomy (OR: 3.894, p = 0.002) were significant risk factors for trifecta failure.

**Table 2:** Univariate and Multivariate Logistic Regression Results in Evaluating Risk Factors for Trifecta Outcome.

Covariates	Univariable analysis			Multivariable analysis		
	OR	95%CI	P	OR	95%CI	P
Age (y)	1.000	0.976-1.025	0.982	0.995	0.969-1.021	0.685
Sex (Male)	0.784	0.398-1.545	0.481	0.509	0.241-1.072	0.076
Tumor Size (cm)	1.987	1.201-3.289	0.008	2.144	1.231-3.736	0.007
Preoperative eGFR (mL/min/1.73m^2^)	0.990	0.980-1.001	0.069	0.991	0.980-1.003	0.130
History of Ipsilateral Partial Nephrectomy	3.372	1.487-7.649	0.002	3.894	1.630-9.304	0.002

OR = Odds Ratio; CI = Confidence Interval; eGFR = Estimated Glomerular Filtration Rate

## DISCUSSION

As a well-established treatment modality, RFA has emerged as an effective therapeutic option for renal masses, particularly those smaller than 4 cm in diameter. Numerous studies have validated the efficacy and safety of RFA in treating T1a renal masses. For example, a long-term follow-up study involving 203 patients who underwent RFA reported a median survival time of 7 years, a 5-year survival rate of 80%, and a low incidence of serious complications of only 3.9% (16). Similarly, studies conducted across multiple countries, including the United States, the United Kingdom, China, and Denmark, have reported tumour-related 5-year survival rates exceeding 95% and local progression rates below 6.5% (17–21). Despite these encouraging results, there is a notable paucity of large-sample studies, and few studies have integrated the trifecta outcome concept into the prognostic evaluation of RFA. The Department of Urology at the First Hospital of Peking University is a pioneer in the use of RFA for the treatment of renal masses, supported by an extensive patient population and a comprehensive database. The aim of this study was to leverage the trifecta outcomes to evaluate the prognosis of patients with stage T1a renal masses who underwent RFA at our institution. Furthermore, we seek to identify risk factors influencing the trifecta outcomes, thereby providing evidence-based insights to optimize clinical treatment strategies.

Our findings further validated the safety and efficacy of ultrasound-guided RFA for the treatment of T1a renal masses and demonstrated a lower recurrence rate, incomplete ablation rate, and complication incidence over an extended follow-up period. A comparison with previous studies is summarized in Supplementary Table-1 (16, 19–26). Among the cohort of patients with 270 T1a renal masses treated over the past 7 years, the median tumour size was 1.97 cm, with a recurrence rate of 7.4%, which is in line with previous studies (16, 19–26). Any differences in comparative outcomes may be attributed to the longer follow-up duration, which likely improved the detection of recurrence events. Additionally, our cohort included a greater proportion of patients with a history of ipsilateral partial nephrectomy (11.9%), a subgroup known to have elevated recurrence risks due to potential residual or recurrent disease. The incomplete ablation rate was 7.4%, which is consistent with previous studies (16, 19–26). Differences in incomplete ablation rate may be attributed to the predominant use of CT-guided approaches in previous studies, where CT imaging allowed clearer delineation of tumour margins than did ultrasound, thereby facilitating more precise ablation and reducing the likelihood of incomplete treatment. Furthermore, the greater proportion of endophytic tumours in our cohort (30.0%), which were more challenging to ablate completely due to their proximity to critical structures and heat-sink effects from adjacent vasculature, may have contributed to the observed variability. Notably, the incidence of severe complications was 1.1%, which was lower than that reported in earlier studies (16, 19–26). This enhanced safety profile may result from the real-time visualization capabilities of ultrasound guidance, which facilitate precise probe-tumour positioning and minimize the risk of injury to blood vessels and adjacent structures. Additionally, the application of colour Doppler ultrasound or contrast-enhanced ultrasound guidance has been shown to further reduce bleeding risk during RFA procedures.

**Supplementary Table 1:** Comparative Outcomes of Radiofrequency Ablation for T1a Renal Masses.

Authors	Year	Image Guidance	Tumors (n)	Tumor Size (cm)	Follow-up (months)	Incomplete Ablation (%)	Tumor Recurrence (%)	Severe Complications (%)
Iannuccilli JD, et al. (16)	2015	CT	203	2.5	34.1	5	7.4	3.9
Chang X, et al. (19)	2015	US	134	3.6	67.6	0	1.5	4.4
Zhang F, et al. (20)	2015	CT/US	122	3.4	64.9	4.9	5.7	NA
Liu N, et al. (22)	2017	US	115	NA	77	0	7.0	NA
McClure T, et al. (23)	2018	CT/US	100	2.6	24	5	5	3.5
Marshall HR, et al. (24)	2020	CT/US	125	2.2	62.8	10	6	2.4
Sun Y, et al. (25)	2021	CT/US	103	2.4	12	NA	7.8	0
Bersang AB, et al. (21)	2021	CT	124	2.3	60	2	5	1.7
Abdelsalam ME, et al. (26)	2023	CT	243	2.5	44.4	3.1	4.5	4.1
Our Study	2025	US	270	1.97	35.6	7.4	7.4	1.1

CT = Computed Tomography; US = Ultrasound.

The concept of "trifecta outcomes" has garnered significant attention in recent years as a valuable metric for evaluating postoperative surgical outcomes. In renal oncology, this metric has been widely applied to assess the outcomes of partial and radical nephrectomy, which are conventionally defined as surgical success, functional preservation, and oncological efficacy (10–13). However, prior studies on minimally invasive ablative therapies have focused predominantly on isolated outcomes such as recurrence, incomplete ablation, or severe complications and have rarely integrated these factors; consequently, the trifecta of outcomes has remained underutilized in this context. To address this gap, we adapted the trifecta outcome framework specifically for RFA of T1a renal masses, which is defined as the absence of severe complications, tumour recurrence and incomplete ablation. It not only reflects surgical safety and tumour control but also predicts long-term patient prognosis and impacts patient quality of life (27, 28). As such, it has become an indispensable tool for guiding postoperative treatment and follow-up strategies, ensuring that clinical assessments align closely with patients’ real-world experiences.

In our cohort, the trifecta outcomes were achieved in 84.1% of patients. Notably, this rate surpassed the 77.3% trifecta success rate reported in a prior study of 119 T1a renal masses treated with RFA, which documented a median follow-up of 43 months (29). The observed discrepancy may reflect differences in follow-up duration, as longer surveillance periods increased the likelihood of detecting delayed recurrence or incomplete ablation. Tumour size and a history of ipsilateral partial nephrectomy were identified as independent predictors of trifecta failure. Patients with larger tumours were more likely to experience trifecta failure, which was perhaps not surprising given that larger tumours required broader ablation zones to ensure complete destruction of the tumour tissue. A larger ablation range may cause greater damage to the surrounding tissue, thereby increasing the risk of severe complications. Furthermore, tumour size has been widely recognized as a risk factor for tumour recurrence, with numerous studies establishing a correlation between tumour size and recurrence (30). For example, Johnson Brett's study demonstrated that tumours exceeding 3 cm significantly reduced the 5-year recurrence-free survival rate (31). Our findings suggest that tumour size may also serve as a predictor of incomplete ablation, which is consistent with the observations of Cameron (32), although the underlying mechanism remains to be fully elucidated.

Patients with a history of ipsilateral partial nephrectomy were identified as being at greater risk of trifecta failure. As a more invasive procedure, partial nephrectomy is associated with a greater risk of complications and a subsequent decline in renal function (33, 34). This compromised renal function may increase the risk of severe complications, thereby contributing to trifecta failure. Additionally, partial nephrectomy, despite its relatively high disease-free survival rate (35), might predispose patients requiring subsequent RFA to tumour recurrence, as prior surgery could indicate residual or recurrent disease, thereby contributing to trifecta failure. Clinically, surgeons have adopted more conservative ablation parameters in patients with a history of partial nephrectomy to preserve renal function, which could inadvertently result in a higher incidence of incomplete ablation in this population.

When our findings are contextualized within the broader landscape of renal mass management, it is instructive to compare recurrence rates between ultrasound-guided RFA and partial nephrectomy. In the present study, the tumour recurrence rate following RFA was 7.4% over a median follow-up of 35.6 months. In contrast, prior studies on partial nephrectomy reported lower recurrence rates. For example, a cohort of 110 patients who underwent partial nephrectomy demonstrated a 3.6% local recurrence rate over a mean follow-up of 3.12 years (36). Similarly, another study reported a 2.9% recurrence rate after partial nephrectomy, with a mean follow-up of 31 months (37). This discrepancy may result from inherent differences in therapeutic mechanisms and patient selection. Partial nephrectomy involves direct surgical excision with histopathological margin assessment, potentially achieving more complete tumour removal, particularly for anatomically complex or larger tumours supported by preoperative 3D reconstruction to optimize surgical planning (38). In contrast, RFA relies on thermal energy to ablate tumour tissue, which might be less effective in eradicating microscopic extensions or tumours adjacent to the heat-sink vasculature. Notably, RFA is primarily indicated for T1a renal cell carcinoma, with limited efficacy and safety data for larger tumours. In comparison, partial nephrectomy offers a broader range of indications. Additionally, the precision of electrode placement under ultrasound guidance is highly operator dependent, particularly for endophytic or irregular lesions, which may further compromise ablation outcomes. Additionally, RFA is often reserved for patients who are deemed suboptimal surgical candidates due to comorbidities or compromised renal function, which could introduce selection bias towards higher-risk populations; this highlights the importance of patient selection, as RFA is generally prioritized for elderly or comorbid patients with small, exophytic tumours, whereas younger, healthier individuals or those with complex tumours may benefit more from surgical resection to achieve definitive oncologic control. Notably, in our cohort, 11.9% of patients had a history of ipsilateral partial nephrectomy, a factor independently associated with trifecta failure, further underscoring the complexity of this population. Moreover, RFA has several advantages over partial nephrectomy in reducing procedure-related complications. Unlike partial nephrectomy, RFA avoids the need for renal artery clamping, thereby eliminating the risks of ischemic injury and reperfusion damage. Additionally, the precision of RFA's thermal energy delivery minimized collateral damage to healthy renal parenchyma, preserving the nephron mass and reducing the likelihood of severe renal insufficiency or subsequent dialysis. These benefits are particularly critical for patients with preexisting renal impairment or solitary kidneys, where functional preservation is paramount.

In conclusion, our study highlighted key risk factors influencing trifecta outcomes in patients who underwent RFA for T1a renal masses. These findings underscore the importance of developing personalized treatment strategies to optimize clinical outcomes. Future research could focus on validating these risk factors in larger multicentre cohorts and investigating additional variables affecting the trifecta outcomes of RFA.

Several limitations of this study should be acknowledged. First, its retrospective design and single-centre nature might limit generalizability and introduce selection bias. Second, the median follow-up duration was 35.6 months, and the lack of prospective data precluded definitive causal conclusions regarding risk factors for trifecta failure. Third, the limited number of laparoscopic, CT-guided, and other modality-guided RFA procedures at our center prevented comparative analyses of outcomes across ablation techniques.

## CONCLUSIONS

Ultrasound-guided RFA is a safe and effective treatment for T1a renal masses. Tumour size and a history of ipsilateral partial nephrectomy were associated with trifecta failure.

## Data Availability

The data that support the findings of this study are available from the corresponding author upon reasonable request.
